# Malignant transformation of nevus sebaceous to basal-cell carcinoma: Case series, literature review, and management algorithm

**DOI:** 10.1097/MD.0000000000029988

**Published:** 2022-08-05

**Authors:** Yeon Ji Lee, Hye Ju Han, Dong Yeon Kim, Chang Young Yoo, Jin Soo Lim

**Affiliations:** a Department of Plastic and Reconstructive Surgery, College of Medicine, St. Vincent Hospital, The Catholic University of Korea; b Department of Pathology, College of Medicine, St. Vincent Hospital, The Catholic University of Korea.

**Keywords:** basal-cell carcinoma, malignant degeneration, nevus sebaceous

## Abstract

Nevus sebaceous (NS) is a common congenital hamartoma of the skin composed predominantly of sebaceous glands. Although most NS are benign skin tumors, malignant transformations have been reported. There is still controversy about the lifetime risk of malignant degeneration and precise surgical criteria. This study reports cases of malignant degeneration and suggests a surgical treatment algorithm. The medical records of patients with basal-cell carcinoma (BCC) arising from NS between January 2001 and January 2021 were retrospectively reviewed. Patient demographics including lesion location, and tumor size were investigated. The symptoms, histological findings before and after excision, complications, and recurrence during 2-year follow-up periods were investigated. Ten patients were identified with BCC arising from NS lesions. All patients were female and the mean age was 52.11 years. All patients complained of sudden morphological changes, the most common type being rapid color changes. Two cases had histological findings that showed a miss-match between punch biopsy and excisional biopsy results. No recurrence was detected 2 years after surgeries in any patients. Cases after third stage, especially in over 40 years who report morphologic changes, should undergo total surgical excision as the first approach, with strong suspicion of malignant degeneration.

## 1. Introduction

Nevus sebaceous (NS) is a common congenital hamartoma of the skin composed predominantly of sebaceous glands and often occurs on the scalp, face, and neck.^[1]^ NS typically presents as finely papulated alopecic plaque that is usually pink-orange in color when occurring in infant stage. The second stage, which occurs during pubertal expansion, includes clinical features such as a verrucous, pruritic, and friable nodular lesion that is yellowish or brownish in color.^[2]^

Although most NS are benign skin tumors at the time of discovery, malignant transformation has been documented to occur over 3 evolutionary and overlapping stages. The most common malignant tumor derived from NS is basal-cell carcinoma (BCC), revealing that NS and BCC share deletions of the PTCH tumor suppressor gene on chromosome 9q22.3.^[3,4]^ Reported incidence of malignant transformation to BCC varies from 0.8 to 22%.^[5–7]^ Therefore, when surgeons encounter patients with NS, they should determine whether prophylactic excision is required given the possibility of malignant transformation according to age and morphologic features. However, criteria for recommended surgical treatment differ from study to study.

In a recent clinical study, second-stage NS (or the puberty stage) passes through proliferation with rapid morphological and histological changes, so prophylactic excision is recommended.^[8]^ Another study recommends prophylactic excision in earlier stages because malignancy is possible even among patients in early prepuberty childhood.^[6]^ However, another study argues that prophylactic excision is unnecessary because malignant transformation before puberty is extremely rare.^[9]^

There is still controversy about the lifetime risk of malignant degeneration and precise surgical criteria among different studies. Therefore, this study reports cases of malignant degeneration and presents lifetime risk of malignant degeneration and treatment algorithms including precise surgical criteria to provide surgeons information to determine the best timing for prophylactic excision.

## 2. Methods

The medical records and clinical data of patients treated at St. Vincent Hospital between January 2001 and January 2021 were retrospectively reviewed. After identifying patients diagnosed with BCCs, those who had a congenital history of NS lesions in the same location as the arising BCC were included in this study. All patients who did not have NS lesions before BCC diagnosis were excluded. Patient demographics including age, sex, lesion location, and tumor size were investigated.

Before surgery, patient-reported symptoms were investigated, including lesion changes were recorded. Pathologic confirmation through punch biopsy or incisional biopsy and histological findings were also recorded. After total surgical excision, the final histological finding was confirmed, and consistency between pre- and postoperative pathology was investigated. Additionally, the safety-margin size secured during surgical excision, reconstruction method, and long-term and short-term complications were investigated. Recurrence over the 2-year follow-up period was investigated.

The patients provided informed consent for publication of their cases.

### 2.1. Ethics statement

This study was approved by the Institutional Review Board of Human Research Protection Program. All data were analyzed anonymously and according to the principles outlined in the Declaration of Helsinki 1975 (revised in 2008).

## 3. Results

**Figure 1.**
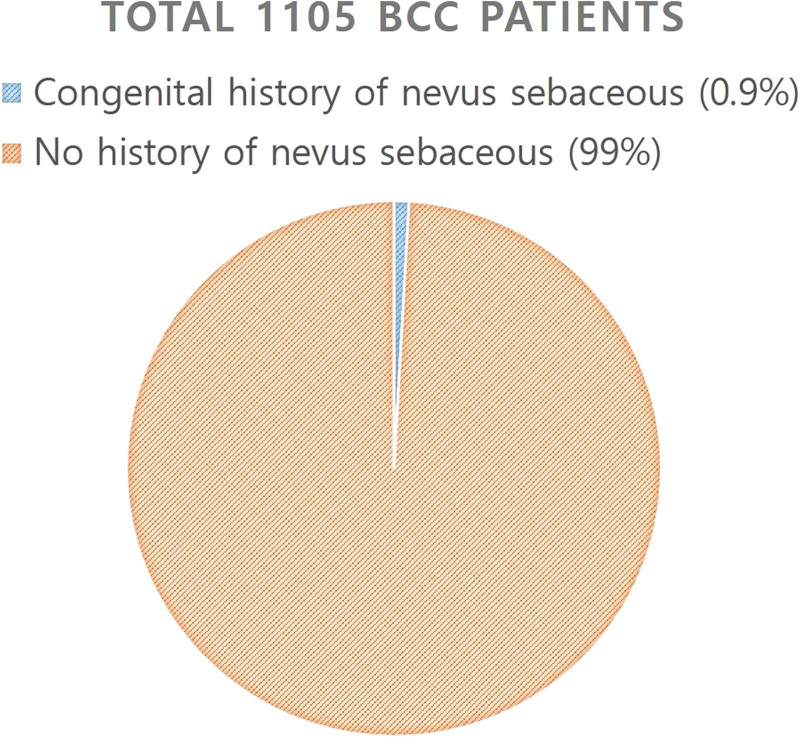
Pie diagram of patients. Of a total of 1105 BCC patients, 10 were found to have developed BCC from the NS. BCC = basal-cell carcinoma, NS = nevus sebaceous.

Between January 2001 and January 2021, of a total of 1105 BCC patients, 10 patients were identified as having BCC arising from NS lesions (Fig. 1). All patients were female with a mean age of 52.11 years. All patients had BCC on the scalp, 4 of which were in the parietal area, 5 were in the temple, and 1 was in the vertex area.

**Figure 2.**
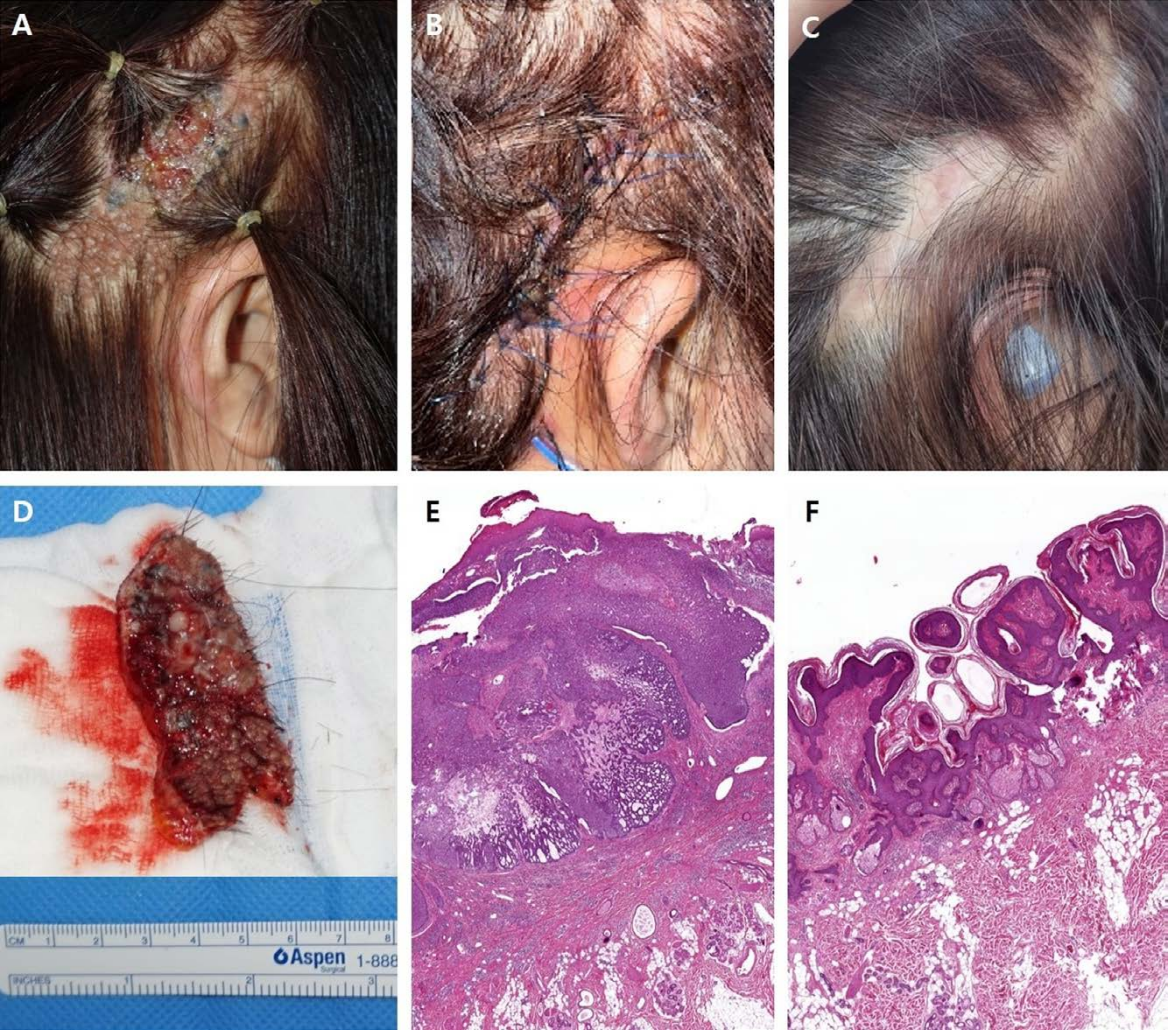
Various morphologic changes of NS. All patients complained of sudden morphological changes, the most common being rapid color change to blackish, brownish, and erythematous lesions. Other symptoms included size expansion, protruding mass, ulceration, nodular surface, itching sensation, and discharge. NS = nevus sebaceous.

All patients complained of sudden morphological changes, the most common being rapid color change to blackish, brownish, and erythematous lesions. Other symptoms included size expansion, protruding mass, ulceration, nodular surface, itching sensation, and discharge (Table 1; Fig. 2A–H).

**Table 1:** Patient demographics and clinical characteristics of 10 cases.

No.	Sex/age	Location	Size	Morphologic change	Initial histologic finding (punch biopsy)	Final histologic finding	Safety margin from the NS margin	Complication	Recurrence (2 yrs F/U)
1	F/49	Scalp (parietal)	3 × 10	Color change (blackish), size expansion, itching sense	Trichoblastoma	BCC	3 mm	Wound disruption Alopecia	No
2	F/42	Scalp (parietal)	2.5 × 8	Color change (blackish), ulcer, size expansion	BCC	BCC	No	Alopecia	No
3	F/56	Scalp (temple)	4 × 8	Color change (brownish), ulcer	BCC	BCC	3 mm	Alopecia	No
4	F/55	Scalp (temple)	3.5 × 6.5	Color change (blackish), nodular surface, size expansion	BCC	BCC	No	No	No
5	F/61	Scalp (vertex)	3.5 × 4	Color change (erythematous), protruding mass, ulcer, Itching sense	–	BCC	No	Alopecia	No
6	F/61	Scalp (parietal)	2 × 8	Color change (erythematous), Ulcer	–	BCC	3 mm	No	No
7	F/46	Scalp (temple)	3 × 7	Itching sense, size expansion	–	BCC	No	Alopecia	No
8	F/70	Scalp (temple)	3 × 5	Color change (blackish), nodular surface	BCC	BCC	3 mm	No	No
9	F/51	Scalp (parietal)	5 × 8	Color change (brownish) Nodular surface	BCC	BCC	No	Alopecia	No
10	F/44	Scalp (temple)	3 × 5	Color change(blackish), Protruding mass, Ulcer	Trichilemmoma	BCC	No	Alopecia	No

Seven patients underwent preoperative punch biopsy. Two cases had histological mismatches between punch biopsy and excisional biopsy results, where punch biopsy identified trichoblastoma and trichilemmoma, but the lesions were ultimately confirmed to be BCCs after total excision.

Four patients received excision with a safety margin that included 3 mm of normal skin around the NS lesion, and 5 patients received surgery with the just the NS margin of NS and no normal safety margin.

Complications were reported in 1 case of wound disruption that healed completely after conservative treatment and in 7 cases with long-term alopecia. No recurrences were detected after 2 years of postsurgery follow-up (Table 1).

### 3.1. Case 1

**Figure 3.**
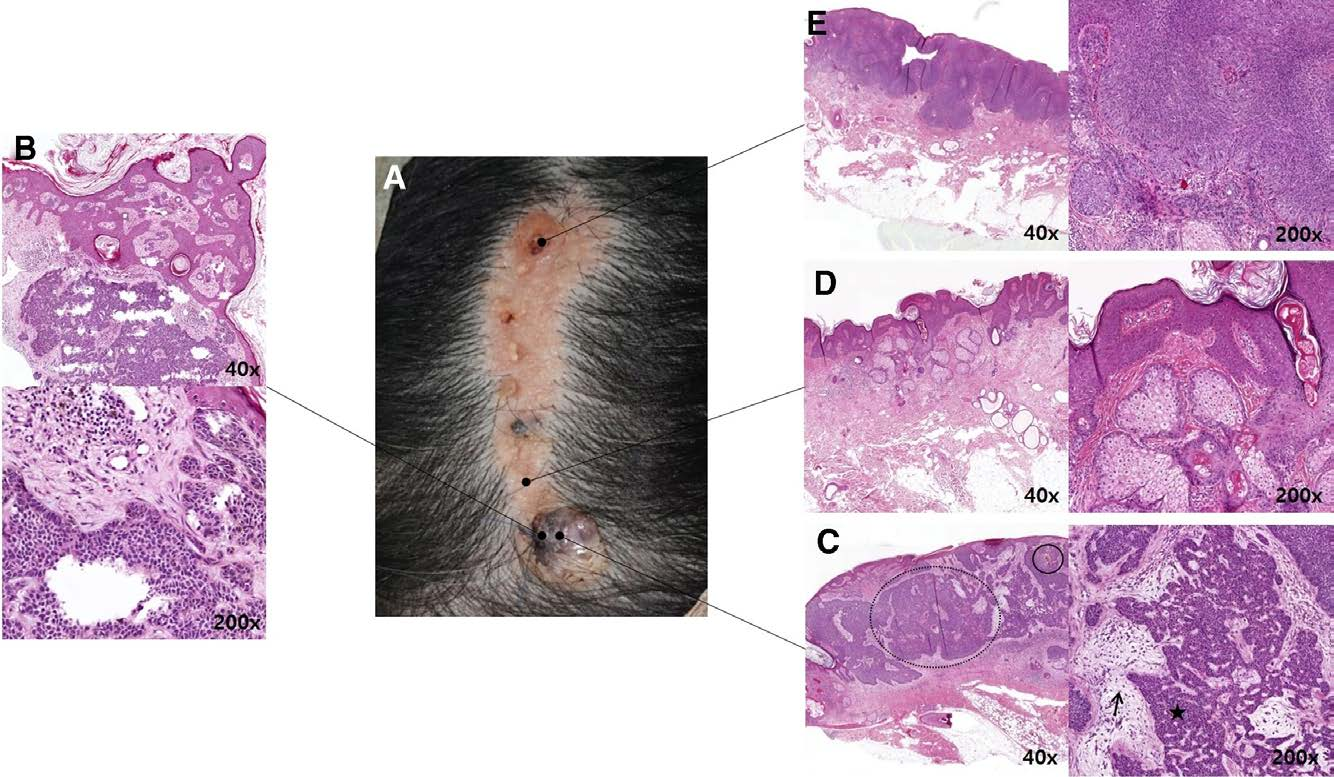
Clinical and histological findings of benign and malignant degeneration from NS. (A) Gross finding of scalp lesion, about 3 cm × 10 cm-sized yellowish linear plaque on left parietal area of scalp with focal blackish nodule palpated on lower portion of the lesion (B); Initial biopsy results of trichoblastoma that arose from NS include a relatively well-circumscribed cell nest in the superficial dermis with peripheral palisading and features of trichogenic follicular papillae (H&E, 40×, 200×) (C); Histologic features of BCC degenerated from NS shows basaloid cell nest (dotted circle), hyperchromatic nuclei(star) and peripheral palisading (arrow) with focal myxoid change and melanin pigments (solid line circle) in surrounding stroma (H&E, 40×, 200×) (D); The nevus sebaceous lesion shows diffuse epidermal papillomatosis and abundant sebaceous glands (H&E, 40×, 200×) (E); Histologic features of a trichilemmoma degenerated from NS shows a folliculocentric tumor composed of basaloid cells with clear cytoplasm, bulbous profile, peripheral palisading, and connection to the epidermis in the background of the nevus sebaceous (H&E, 40×, 200×). BCC = basal-cell carcinoma, NS = nevus sebaceous.

A 61-year-old female patient had an approximately 3 cm × 10 cm-sized yellowish linear plaque on the left parietal area of the scalp with a focal blackish palpated nodule on the lower portion of the lesion (Fig. 3A). Although the onset of color change was uncertain, she complained of sudden size expansion and itching. Pathologic confirmation using 6-mm punch biopsy was performed prior to surgical excision, and the punch biopsy specimen was intended to include the lesion of blackish color change where malignant degeneration is most suspected. The initial punch biopsy results indicated trichoblastoma that arose from a NS. The lesion showed a relatively well-circumscribed cell nest in the superficial dermis with peripheral palisading and features of trichogenic follicular papillae (Fig. 3B).

We performed extensive resection with just the NS margin without a normal-skin safety margin, which left a full-thickness scalp defect. After confirming that no more NS lesions remained, coverage operation were performed to cover this defect. We planned local flap coverage and closure with a metal stapler.

In contrast to the initial biopsy, the pathologic feature of the surgical excision revealed secondary tumors including 2 foci of BCC and a benign tumor, most likely trichilemmoma, in the background of NS. The lesion BCC showed a basaloid cell nest, hyperchromatic nuclei, and peripheral palisading with focal myxoid change and melanin pigments in the surrounding stroma (Fig. 3C). The NS lesion showed a diffuse epidermal papillomatosis and abundant sebaceous glands (Fig. 3D). Histologic feature of a trichilemmoma shows a folliculocentric tumor composed of basaloid cells with clear cytoplasm, bulbous profile, peripheral palisading, and connection to the epidermis in the background of NS (Fig. 3E). There was no lymphovascular invasion or resected margin involvement.

After surgical excision, mild wound disruption was detected that healed with conservative wound care without secondary surgery. Six months after surgery, the patient complained about the scar quality and alopecia over a 1.5-mm area. Because the scar and linear alopecia were well covered with hair, additional revision surgery was not performed. Recurrence was not observed during the 2-year follow-up period.

### 3.2. Case 2

**Figure 4.**
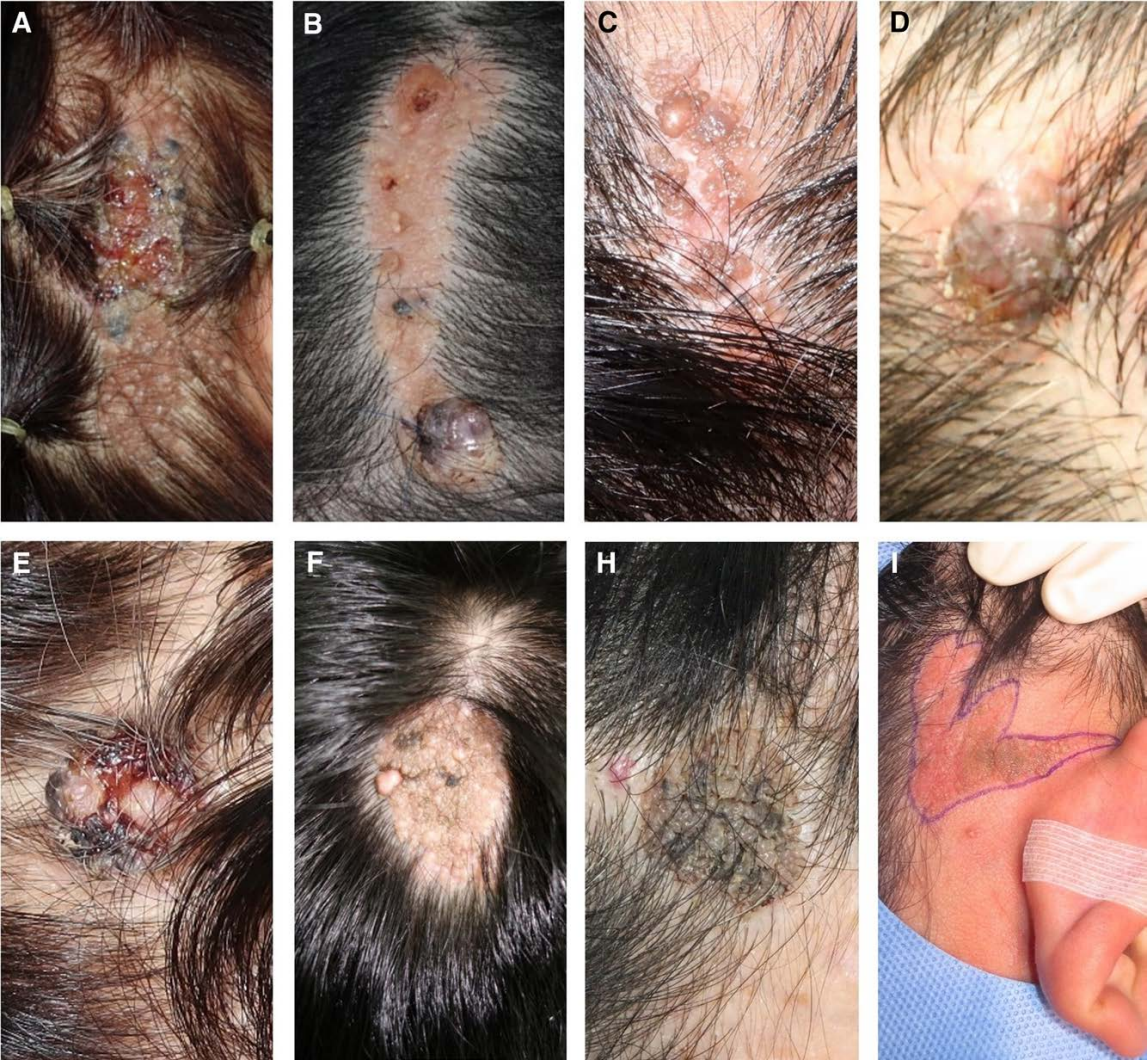
Clinical findings of malignant degeneration to BCC from NS. (A) Initial lesion, multiple ulcers, and blackish nodules on 2 cm × 8 cm-sized linear brownish patch (B); immediate postoperative clinical photo (C); 1-year postoperative clinical photo (D); pathologic specimen excised with 5-mm safety margin (E); Histologic features of BCC (H&E, 40×) (F); histologic features of NS (H&E, 40×). BCC = basal-cell carcinoma, NS = nevus sebaceous.

A 62-year-old female patient visited our clinic with recent blackish color change of a congenital scalp mass. Initially, multiple ulcers and blackish nodules on a 2 cm × 8 cm-sized linear brownish patch spread over her right temporal area (Fig. 4A). An initial punch biopsy performed 4 years ago confirmed desmoplastic trichilemmoma, a benign lesion. We performed extensive resection with a 3-mm safety margin, leaving a full-thickness scalp defect. To cover this defect, we performed local flap coverage with nylon sutures (Fig. 4B). One year after surgery, the wound was stable without evidence of recurrence (Fig. 4C). Biopsy results confirmed BCC secondary to NS (Fig. 4D). The BCC showed a basaloid epithelial tumor with peripheral palisading at the adjacent tumor stroma (Fig. 4E). Around the BCC, the NS lesion shows papillomatosis and sebaceous hyperplasia (Fig. 4F).

## 4. Discussion

The natural history of NS involves differentiation through 3 evolutionary and overlapping stages.^[2]^ In the first stage, the infancy and childhood stage, NS clinically presents as a round, oval, or linear smooth, yellowish patch or plaque of alopecia.^[10,11]^ The incipient sebaceous glands appear as bright yellow dots in dermoscopy, which is the clinical diagnostic point for differentiating sebaceous nevus from aplasia cutis congenital.^[12]^ At the second stage or the puberty stage, dermoscopic findings include yellowish globules with a cobblestone pattern, and numerous, hyperplastic sebaceous glands in histopathology.^[10,13]^ The third stage, or post pubertal stage, presents as yellowish-brown globules, fissures, and ridges arranged in a cerebriform pattern, dermoscopically.^[14]^

According to studies of histological degeneration of NS, the 2 most common benign tumors arising from NS are trichoblastoma and syringocystadenoma papilliferum, and the most common malignant tumor is BCC.^[7,14,15]^ Malignant transformation usually develops at the third stage and even benign tumors have been observed before the third stage.

BCCs arising from NSs typically occur in adults older than 40 years.^[7]^ Risks factors for malignant transformation are older age, tumor presentation on scalp, and positive familial BCC history.^[16]^ Another study reported that positive familial BCC history, human papilloma virus seropositivity, ultraviolet exposure, and fair skin type (Fitzpatrick skin types I and II) can accelerate malignant degeneration.^[17]^ It has also been suggested that HRAS motion is related to malignant transformation.^[18]^

The malignant degeneration process is often accompanied by rapid morphological changes and other symptoms. Morphological changes and symptoms reported in the literature include pigmented color change, protruding mass, ulceration, size change, and itching.^[19,20]^ In this study, all patients complained of sudden morphological changes, the most common type being rapid color change. Other symptoms included protruding mass, ulceration, itching sensation, and discharge, and these morphological transformations often occurred simultaneously. It should be noted that a few cases of malignant transformation occurred where only erythematous change was present and not a blackish or brownish color change. Therefore, surgeons should be suspicious of malignant transformation for all morphological changes, including atypical morphological changes.

In cases where pathologic diagnosis was confirmed through punch or incisional biopsy, there is a possibility of misdiagnosis because various histological transformations coexist within a single lesion that has also undergone malignant degeneration. As malignant degeneration proceeds through 3 evolutionary and overlapping stages, histological findings of sebaceous carcinoma, BCC, trichoadenoma, trichoblastoma, and syringocystadenoma papilliferum can be simultaneously detected.^[21]^ In this study, 2 such cases were detected, in which trichoblastoma and syringocystadenoma papilliferum were identified and BCCs were masked in the punched biopsy performed prior to surgery, but BCC was eventually diagnosed through total excision surgery of the whole lesion. Therefore, it is recommended that the surgeon obtain a sufficiently large specimen or 2 to 3 specimen pieces within the part of the lesion containing the morphologic change for a precise pathologic diagnosis when performing punch or incisional biopsy prior to surgery. Moreover, it is not easy to differentially histologically diagnose BCC from a benign trichoblastoma, resulting in misdiagnosis. To reduce the possibility of misdiagnosis, immunohistochemistry using markers such as laminin5-c2, nestin, CD10, cytokeratin 15, cytokeratin 19, and cytokeratin 20 have been used.^[22,23]^ Recently, diagnostic accuracy has been improved by using stem cell markers such as pleckstrin homology-like domain, family A, member 1 (PHLDA1).^[24,25]^

According to results from this study and our literature review, treatment criteria should be considered according to patient age and the precise timing of prophylactic excision. Prophylactic excision during the first stage is still controversial. In most large-scale studies, prophylactic excision is not recommended because malignant degeneration is extremely rare before the second stage.^[7,15,26]^ However, aggressive malignant degeneration in early childhood, such as squamous-cell carcinoma, has occasionally been reported.^[27]^ In an 18-year review of 631 patients, the youngest age at BCC development was 9.7 years, accounting for 0.8% of total incidence.^[6]^ Therefore, when a physician clinically suspects malignancy, sufficient evaluation should be conducted regardless of age. Malignancy has not yet been reported in cases of uniform appearance in the first stage. In cases of surface irregularity or irregularly pigmented lesions, a tissue biopsy should be performed for suspected malignancy, and information should be provided so that the surgeon, parent, and patient can actively participate in decision-making about surgery.^[6]^

Prophylactic excision at the puberty stage is recommend according to standard surgical treatment because NSs are known to pass through a proliferation phase with rapid morphological and histological changes at this stage.^[9]^ However, considering the very low incidence of malignant transformation at this stage according to some studies^[6,7]^ and complications such as alopecia after widely applied prophylactic excision surgery in adolescent patients, prophylactic excision needs to be carefully considered.

In this study, all patients with malignant degeneration to BCC were older than 40 years. In a review of 569 cases, BCCs arising from NS were reported to occurred in adults older than 40 years.^[7]^ Furthermore, as mentioned above, histopathologic diagnosis by punched or incisional biopsy could be missed due to coexistence of various histological findings. Therefore, in patients over 40 years who complain of NS accompanied by morphological change, total excision surgery is the most effective for accurate diagnosis and reduction of diagnosis timing.

**Figure 5.**
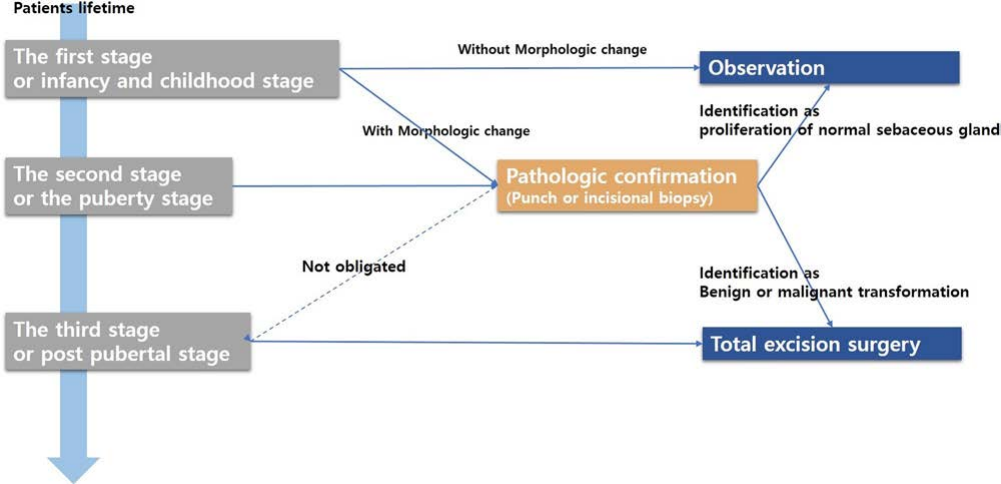
Treatment algorithm. During the first- and second-stage, patients without any morphologic suspicions do not require prophylactic excision and any patients with suspicious morphologic change are recommend to get pathologic confirmation prior to prophylactic excision. After third state NS, especially for patients over 40 years who report morphologic change, total surgical excision is the first choice of treatment due to strong suspicion of malignant degeneration. NS = nevus sebaceous.

In summary, at the first and second stages, patients without any morphologic suspicions do not need to undergo prophylactic excision, and any patients with suspicious morphologic change are recommending to receive pathologic confirmation through punch or incisional biopsy. The current recommendation is to obtain a sufficiently large specimen or 2 to 3 pieces of specimens within the lesion area containing the morphologic change. For patients older than 40 years, pathologic confirmation does not necessarily need to precede excision surgery. It is thought to be effective that total surgical excision is considered a first choice of treatment approach (Fig. 5).

When performing total excision surgery, to obtain negative margins, it has been recommended to secure a safety margin that includes a 3- to 5-mm margin of normal skin around each NS from NS lesion in general.^[6,28]^ However, recurrences were not detected even in patients who underwent surgery with just the NS margin without a normal-skin safety margin. Therefore, a 3- to 5-mm safety margin of normal skin is not essential. Thus, the safety margin can be determined flexibly for cases where aggressive reconstruction is required or where a large alopecia is predicted after an extensive excision.

Although this study was based on gross and histological analysis of the case of BCC from NS lesion, there is a limitation in that it lacks genetic analysis as a confirmatory test. In addition, our study found conflicting results with the previous report that BCC appeared predominantly in males,^[7]^ and therefore, a larger-scale further study is needed.

## 5. Conclusion

Patients with first or second-stage lesions without any morphologic suspicions do not need to receive prophylactic excision, and any patients with suspicious morphologic change are recommend to receive pathologic confirmation prior to prophylactic excision. After NS reaches the third state, especially in over 40 years who report morphologic change, total surgical excision is the first treatment choice because of strong suspicion of malignant degeneration.

## References

[1] Mehregan AH Pinkus H. Life history of organoid nevi. Special reference to nevus sebaceus of Jadassohn. Arch Dermatol. 1965;91:574–88. 14295518 10.1001/archderm.1965.01600120006002

[2] Moody MN Landau JM Goldberg LH. Nevus sebaceous revisited. Pediatr Dermatol. 2012;29:15–23. 21995782 10.1111/j.1525-1470.2011.01562.x

[3] Xin H Matt D Qin JZ. The sebaceous nevus: a nevus with deletions of the PTCH gene. Cancer Res. 1999;59:1834–6. 10213487

[4] Lu Y Zhu HG Ye WM. A new mutation of PTCH gene in a Chinese family with nevoid basal cell carcinoma syndrome. Chin Med J (Engl). 2008;121:118–21. 18272036

[5] Constant E Davis DG. The premalignant nature of the sebaceous nevus of Jadassohn. Plast Reconstr Surg. 1972;50:257–9. 5050785 10.1097/00006534-197209000-00010

[6] Rosen H Schmidt B Lam HP. Management of nevus sebaceous and the risk of Basal cell carcinoma: an 18-year review. Pediatr Dermatol. 2009;26:676–81. 19686305 10.1111/j.1525-1470.2009.00939.x

[7] Barankin B Shum D Guenther L. Tumors arising in nevus sebaceus: a study of 596 cases. J Am Acad Dermatol. 2001;45:792–3; author reply 794. 10.1067/mjd.2001.117240 11606940

[8] Barkham MC White N Brundler MA. Should naevus sebaceus be excised prophylactically? A clinical audit. J Plast Reconstr Aesthet Surg. 2007;60:1269–70. 17707706 10.1016/j.bjps.2007.03.038

[9] Santibanez-Gallerani A Marshall D Duarte AM. Should nevus sebaceus of Jadassohn in children be excised? A study of 757 cases, and literature review. J Craniofac Surg. 2003;14:658–60. 14501324 10.1097/00001665-200309000-00010

[10] Donati A Cavelier-Balloy B Reygagne P. Histologic correlation of dermoscopy findings in a sebaceous nevus. Cutis. 2015;96:E8–9. 26761943

[11] Ankad BS Beergouder SL Domble V. Trichoscopy: the best auxiliary tool in the evaluation of nevus sebaceous. Int J Trichology 2016;8:5–10. 27127368 10.4103/0974-7753.179394 PMC4830180

[12] Neri I Savoia F Giacomini F. Usefulness of dermatoscopy for the early diagnosis of sebaceous naevus and differentiation from aplasia cutis congenita. Clin Exp Dermatol. 2009;34:e50–2. 19438569 10.1111/j.1365-2230.2008.03189.x

[13] Zaballos P Gomez-Martin I Martin JM. Dermoscopy of adnexal tumors. Dermatol Clin. 2018;36:397–412. 30201149 10.1016/j.det.2018.05.007

[14] Zaballos P Serrano P Flores G. Dermoscopy of tumours arising in naevus sebaceous: a morphological study of 58 cases. J Eur Acad Dermatol Venereol. 2015;29:2231–7. 26300536 10.1111/jdv.13226

[15] Idriss MH Elston DM. Secondary neoplasms associated with nevus sebaceus of Jadassohn: a study of 707 cases. J Am Acad Dermatol. 2014;70:332–7. 24268309 10.1016/j.jaad.2013.10.004

[16] Chinem VP Miot HA. Epidemiology of basal cell carcinoma. An Bras Dermatol 2011;86:292–305. 21603813 10.1590/s0365-05962011000200013

[17] Paolini F Carbone A Benevolo M. Human papillomaviruses, p16INK4a and Akt expression in basal cell carcinoma. J Exp Clin Cancer Res. 2011;30:108. 22082146 10.1186/1756-9966-30-108 PMC3271997

[18] Cranwell WC Walsh M Winship I. Phacomatosis pigmentokeratotica: Postzygotic HRAS mutation with malignant degeneration of the sebaceous naevus. Australas J Dermatol. 2019;60:e245–e6. 30767209 10.1111/ajd.13007

[19] Enei ML Paschoal FM Valdes G. Basal cell carcinoma appearing in a facial nevus sebaceous of Jadassohn: dermoscopic features. An Bras Dermatol 2012;87:640–2. 22892785 10.1590/s0365-05962012000400023

[20] Paninson B Trope BM Moschini JC. Basal cell carcinoma on a nevus sebaceous of Jadassohn: a case report. J Clin Aesthet Dermatol 2019;12:40–3. PMC6440703 30988872

[21] Miller CJ Ioffreda MD Billingsley EM. Sebaceous carcinoma, basal cell carcinoma, trichoadenoma, trichoblastoma, and syringocystadenoma papilliferum arising within a nevus sebaceus. Dermatol Surg. 2004;30(12 Pt 2):1546–9. 15606837 10.1111/j.1524-4725.2004.30552.x

[22] Sellheyer K Krahl D. PHLDA1 (TDAG51) is a follicular stem cell marker and differentiates between morphoeic basal cell carcinoma and desmoplastic trichoepithelioma. Br J Dermatol. 2011;164:141–7. 20846311 10.1111/j.1365-2133.2010.10045.x

[23] Sellheyer K Nelson P. Follicular stem cell marker PHLDA1 (TDAG51) is superior to cytokeratin-20 in differentiating between trichoepithelioma and basal cell carcinoma in small biopsy specimens. J Cutan Pathol. 2011;38:542–50. 21352265 10.1111/j.1600-0560.2011.01693.x

[24] Sellheyer K Cribier B Nelson P. Basaloid tumors in nevus sebaceus revisited: the follicular stem cell marker PHLDA1 (TDAG51) indicates that most are basal cell carcinomas and not trichoblastomas. J Cutan Pathol. 2013;40:455–62. 23489134 10.1111/cup.12107

[25] Yeh I McCalmont TH LeBoit PE. Differential expression of PHLDA1 (TDAG51) in basal cell carcinoma and trichoepithelioma. Br J Dermatol. 2012;167:1106–10. 22958125 10.1111/j.1365-2133.2012.11165.x

[26] de la Luz Orozco-Covarrubias M Tamayo-Sanchez L Duran-McKinster C. Malignant cutaneous tumors in children. Twenty years of experience at a large pediatric hospital. J Am Acad Dermatol. 1994;30(2 Pt 1):243–9. 8288784

[27] Taher M Feibleman C Bennett R. Squamous cell carcinoma arising in a nevus sebaceous of Jadassohn in a 9-year-old girl: treatment using Mohs micrographic surgery with literature review. Dermatol Surg. 2010;36:1203–8. 20533930 10.1111/j.1524-4725.2010.01611.x

[28] Depeyre A Barthelemy I Dechelotte P. A case of basaloid degeneration of nevus sebaceous during childhood: should nevus sebaceous be excised or followed up? Facial Plast Surg. 2016;32:576–7. 27680530 10.1055/s-0036-1584556

